# Lack of detection of human papillomavirus DNA in prostate carcinomas in patients from northeastern Brazil

**DOI:** 10.1590/1678-4685-GMB-2015-0122

**Published:** 2016

**Authors:** Ari P. Araujo-Neto, Hygor Ferreira-Fernandes, Carolina M.M. Amaral, Lina G. Santos, Antônio C. Freitas, Jacinto C. Silva-Neto, Juan A. Rey, Rommel R. Burbano, Benedito B. da Silva, France K.N. Yoshioka, Giovanny R. Pinto

**Affiliations:** 1 Laboratório de Genética e Biologia Molecular, Universidade Federal de Piauí, Parnaíba, PI, Brazil; 2 Laboratório de Estudos Moleculares e Terapia Experimental, Departamento de Genética, Centro de Biociências, Universidade Federal de Pernambuco, Recife, PE, Brazil; 3 Faculdade de Medicina, Universidade Federal do Piauí, Teresina, PI, Brazil; 4 Laboratório de Pesquisas Citológica e Molecular, Departamento de Histologia e Embriologia, Centro de Biociências, Universidade Federal de Pernambuco, Recife, PE, Brazil; 5 Molecular Oncogenetics Laboratory, Unidad de Investigación, Hospital Universitario La Paz, Paseo de la Castellana, Madrid, Spain; 6 Laboratório de Citogenética Humana, Instituto de Ciências Biológicas, Universidade Federal de Pará, Belém, PA, Brazil

**Keywords:** human papillomavirus, prostate cancer, prevalence, HPV PCR detection, molecular epidemiology

## Abstract

Prostate cancer is the second most common cancer among men in western populations, and despite its high mortality, its etiology remains unknown. Inflammatory processes are related to the etiology of various types of tumors, and prostate inflammation, in particular, has been associated with prostate cancer carcinogenesis and progression. Human papillomavirus (HPV) is associated with benign and malignant lesions in the anogenital tract of both females and males. The possible role of HPV in prostate carcinogenesis is a subject of great controversy. In this study, we aimed to examine the prevalence of HPV infections in prostate carcinomas of patients from northeastern Brazil. This study included 104 tissue samples from primary prostate carcinoma cases. HPV DNA was purified and then amplified using MY09/11 and GP5+/GP6+ degenerate primer sets that detect a wide range of HPV types, and with specific PCR primers sets for E6 and E7 HPV regions to detect HPV 16. None of the samples showed amplification products of HPV DNA for primer sets MY09/11 and GP5+/GP6+, or the specific primer set for the E6 and E7 HPV regions. HPV infection, thus, does not seem to be one of the causes of prostate cancer in the population studied.

## Introduction

Prostate cancer is the second most common cancer among men in western populations (Siegel *et al.*, 2014). In Brazil, the National Cancer Institute estimated that approximately 69,000 new cases of this disease will be diagnosed in 2015, representing an increase of approximately 24% compared to 2010 (Facina, 2014). Additionally, the mortality rate of prostate cancer has been showing a worrying rise in all regions of Brazil, especially in the Northeast, where the rate increased from 3.8 in 1980 to 14.3 deaths per 100,000 men in 2010 (Conceição *et al.*, 2014). Since age is the main risk factor associated with prostate cancer, the increase in incidence and mortality of this malignancy can be attributed to increased life expectancy. In Brazil, the share of seniors is expected to increase from 6.9% in 2010 to 22.5% in 2050 (Pew Research Center, 2014). Thus, the development of strategies to control age-associated diseases, such as prostate cancer, is warranted. However, the limited knowledge regarding the etiology of prostate cancer is one of the main challenges associated with the development of preventive measures against this disease (Conceição *et al.*, 2014).

Inflammatory processes are related to the etiology of various types of tumors, and prostate inflammation, in particular, has been associated with prostate cancer carcinogenesis and progression. Infections, especially sexually transmitted ones and those that reach the urogenital tract, are possible causes of intraprostatic inflammation. Infectious organisms are essential for the maintenance of local inflammatory processes, which can cause cellular changes responsible for genetic and epigenetic changes that lead to cell transformation (Sfanos *et al.*, 2013). In this context, special emphasis is given to human papillomavirus (HPV). HPV is considered to be the most common cause of sexually transmitted infections, which are implicated in about 5% of all malignancies (Heidegger *et al.*, 2015). The carcinogenesis of HPV depends on the integration of HPV DNA into the host genome and expression of the oncogenic viral proteins E6 and E7, which inhibit tumor suppressor proteins p53 and pRb, leading to increasing genomic instability, accumulation of oncogene mutations, further loss of cell-growth control, and ultimately cancer (Heidegger *et al.*, 2015).

Interest in the study of HPV has grown steadily since HPV DNA sequences were first detected in biopsies of anogenital cancer. Epidemiological evidence shows that men who develop anal cancer have a higher risk of developing prostate cancer (Rabkin *et al.*, 1992). Previous and recent studies using serological and molecular technologies have suggested an association between infection with high-risk oncogenic HPV and prostate cancer (McNicol and Dodd, 1990; Anwar *et al.*, 1992; Chen *et al.*, 2011; Singh *et al.*, 2015), but the results are still inconclusive, mainly because there is also evidence of the absence of this association (Effert *et al.*, 1992; Bergh *et al.*, 2007; Gazzaz and Mosli, 2009;Yow *et al.*, 2014)

The prevalence of HPV DNA in tumor tissue of patients with prostate cancer has been poorly investigated in Brazil (Silvestre *et al.*, 2009). Therefore, the present study examined for the first time the prevalence of HPV infections in prostate carcinomas of patients from northeastern Brazil.

## Subjects and Methods

This single-center study included 104 patients admitted to the Hospital São Marcos in Teresina, State of Piauí, in the northeastern region of Brazil. The patients were consecutively diagnosed with prostate cancer, with histopathological confirmation between August 2011 and July 2013. All patients enrolled in the study underwent radical prostatectomy as a first treatment option, and fresh frozen specimens of primary prostate tumors were provided by the Pathology Department of the Hospital which were preserved in RNAlater solution (Applied Biosystems, Inc. Foster City, CA) and stored at −20 °C until analysis date. The individuals signed informed consent forms, and the procedures were approved by the Universidade Federal do Piauí Ethics Committee in Research.

For DNA extraction, samples of tumor fragments (approximately 20 mg) were mechanically homogenized (handheld homogenizer, model D-130, Wiggenhause SDN BHD) in nuclear lysis solution (Wizard® Genomic Kit, Promega Inc., USA) and thereafter incubated with 20 mg/ml proteinase K at 65 °C for 12 h or until the fragment was completely digested. Subsequently, proteinase K was inactivated at 95 °C for 15 min and the total product was used for DNA extraction in accordance with the recommendations of the kit manufacturer.

After isolation, the DNA concentration was determined using a Biospec-nano spectrophotometer (Shimadzu, Japan). The DNA obtained from the samples was submitted for amplification of the human *MDM2* gene (Xiao *et al.*, 2010) to verify integrity and quantity of DNA in the samples. Table 1 shows the primer sequences and protocols used to amplify HPV DNA sequences.

**Table 1:** Primers used for amplification of the region of interest, and size of the PCR product.

Primers	Base sequence (5'-3')	T^1^	Size	Product (bp)^2^
MDM2 – F	GATTTCGGACGGCTCTCGCGG	66,5° C	21	121
MDM2 – R	CATCCGGACCTCCCGCGCTG	66,5° C	20	
MY09	CGTCC(AC)A(AG)(AG)GGA(T)ACTGATC	55° C	23	450
MY11	GC(AC)CAGGG(AT)CATAA(CT)AATGG	55° C	23	
GP5+	TTTGTTACTGTGGTAGATACTAC	45° C	23	150
GP6+	GAAAAATAAACTGTAAATCATATTC	45° C	25	
Pr1 (HPV-16)	TCAAAAGCCACTGTGTCCTGA	57° C	21	119
Pr2 (HPV-16)	CGTGTTCTTGATGATCTGCAA	57° C	21	

1 T: annealing temperature;

2 bp: base pairs

HPV DNA detection was performed by PCR amplification using the standard nested PCR approach with the MY09/11 primer set (outer primers) and the GP5+/6+ primer set (inner primers), which are degenerate primer sets that detect a wide range of HPV types (Aghakhani *et al.*, 2011). Additionally, in an attempt to increase detection rate of HPV, a second inner primer set was used (Pr1/Pr2) for amplification of specific sequences of the E6 and E7 regions of HPV 16 (Karlsen*et al.*, 1996). The cycling conditions were 94 °C for 3 min followed by 34 cycles of 94 °C for 30 s, 55 °C (for MY09/11), 45 °C (for GP5+/6+) or 57 °C (for Pr1/Pr2) for 1 min and 72 °C for 1 min, with a final extension step at 72 °C for 10 min. The reaction mix was prepared with 4 μL DNA (200 ng), 1.6 μM of each primer and 12.5 μL MasterMix, in a total volume of 25 μL. The PCR products were visualized on a 2% agarose gel with ethidium bromide under UV light. Each positive sample was then bidirectionally sequenced using the BigDye^®^ Terminator v.3.1 Cycle Sequencing Kit.

Each batch of samples included negative controls without a DNA template, and a positive control, which consisted of a sample of DNA from uterine cervix cells (200 ng) known to be infected with HPV.

## Results

One hundred and four prostate carcinoma samples were analyzed from patients aged between 45 and 90 years (mean = 68 ± 8.5 years). Tumors were staged using standard criteria by the Gleason score and TNM staging system, according to the 7th revision of the TNM classification, 2010. No sample had a Gleason score lower than 6. Table 2 shows the cases along with their clinico-pathological characteristics. Because this study had a retrospective design, complete information was lacking for some patients due to a lack of information about the registration of clinical pathologists.

**Table 2:** Clinico-pathological characteristics of the samples

Variable	Prostatic tumors (n = 104)
Mean age (SD)	68 ± 8.5
Gleason Grade (n = 97)	
Low (Gleason 2-6)	6.2%
Moderate (Gleason 7)	60.8%
High (Gleason 8-10)	33.0%
Tumor extension (n = 96)	
T1	5.2%
T2	21.9%
T3	64.6%
T4	8.3%
Regional lymph nodes (n = 74)	
Absent	85.1%
Present	14.9%
Distant metastasis (n = 59)	
Absent	86.4%
Present	13.6%
HPV DNA (n = 104)	
Absent	100%
Present	0%

The success rate for amplification using *MDM2* gene primers was 100%, indicating that a large quantity of DNA was extracted from the tumors (ranging from 410 to 1880 ng/μL, Figures S1 andS2). However, none of the samples showed amplification products of HPV DNA for primer sets MY09/MY11 and GP5/GP6, or the specific primer set for the E6 and E7 HPV regions. In an attempt to reach a higher assay sensibility, the analyses were repeated using a GP5/GP6 auto-nested PCR approach, as described by Remmerbach *et al.* (2004). However again, all samples tested negative for HPV DNA.

## Discussion

Since 1990, studies in several countries have attempted to detect HPV DNA in prostate carcinomas using different detection methods. Some studies suggested that prostate carcinomas are related to HPV infection and have shown the presence of HPV DNA to varying degrees (from 2 to 100%), but even after several years of HPV DNA analysis in benign and malignant prostate samples, it is still unclear if HPV causes prostate carcinogenesis (Heidegger *et al.*, 2015).

McNicol and Dodd (1990) were the first to demonstrate HPV genomic sequences in prostate tissues. They found that 14 of 15 (93.3%) benign prostatic hyperplasia (BPH) samples from Canadian patients, and all four carcinomas (100%), tested positive for HPV 16. Subsequent studies, such as the one by Anwar *et al.* (1992), provided further evidence to support a relationship between HPV and prostate cancer. According to their results, HPV DNA was present in 28 (41%) out of 68 prostate carcinomas cases from Japanese patients and was not detected in any of the normal or hyperplastic prostatic tissues (10 each), which is consistent with recent findings in other populations, such as the ones from Greece and India (Michopoulou *et al.*, 2014; Singh *et al.*, 2015).

In contrast, several studies have found that HPV DNA is not preferentially associated with either BPH or prostate cancer. Tachezy*et al.* (2012) found no association between the persistence of HPV infection and the risk of developing prostate cancer, reporting the same prevalence of HPV DNA (2%) in 210 samples of tissues from 95 patients with BPH and in 90 samples of 51 patients with prostatic cancer. Other studies have detected HPV DNA sequences in approximately 12–65% of malignant and in 5–48% of benign prostatic samples, and no statistically significant difference was shown (Carozzi *et al.*, 2004;Aghakhani *et al.*, 2011;Chen *et al.*, 2011; Ghasemian *et al.*, 2013). Based on these findings, it can be concluded that the prostate gland is a potential reservoir for transmission of HPV types with oncogenic potential. However, the HPV DNA findings in prostate tissue do not necessarily reflect active infection, or that the prostatic disease was caused by HPV; they could very well represent transient infections. Thus, while the prostate may act as a site for HPV replication, it is unlikely that HPV is directly involved in the transformation of prostatic cells (Dodd *et al.*, 1993;Aghakhani *et al.*, 2011). On the other hand, the presence of high-risk HPV DNA in subsets of prostate cancers shows that at least a proportion of prostate cancers may be related to HPV infection.

In this study we were unable to identify the presence of HPV DNA in any of the 104 samples of prostate carcinoma in patients from northeastern Brazil. Our results are consistent with other previously and recently published reports. Effert *et al.* (1992) analyzed 30 paraffin-embedded prostate adenocarcinomas for the presence of HPV 16- or HPV 18-specific sequences by differential PCR (D-PCR) and Southern blot analysis. Despite the high sensitivity of their analytical technique, they found no evidence of HPV DNA of either type in any of the 30 primary prostate cancers. Furthermore,Bergh *et al.* (2007), in an investigation of 201 prostate tissue samples of patients with BPH that later progressed to prostate cancer and of 201 matched controls, found that all samples tested were negative for HPV, thus making it an unlikely contributing factor for subsequent cancer development. Gazzaz and Mosli (2009) did not detect HPV DNA by hybrid capture 2 technology among 56 tested biopsies categorized into a benign lesion pattern of prostatic hyperplasia, malignant, and inflammatory disorders. Yow*et al.* (2014) achieved the same result after analyzing 195 archival paraffin-embedded tissue blocks by PCR.

Inconsistent results may be due to technical difficulties in HPV DNA detection. There are several possible reasons for false-positive or false-negative results, most notably PCR contamination, degradation, or cross-contamination of DNA, and tumor samples that are not representative (Terris and Peehl, 1997; Adami *et al.*, 2003; Gazzaz and Mosli, 2009). Moreover, considering that HPV presence is strongly associated with high-risk sexual behavior, including a history of sexually transmitted diseases (STDs), the number of sexual partners, and age of first sexual activities, a comparison of cancers from patients with such sexual activity *vs.*those who did not engage in high-risk behavior may have shown different results.

The distribution of HPV varies among different populations, and infection rates are influenced by geography, age, sexual history, coinfections, immune status, and genetic factors (Vesco *et al.*, 2011). In Brazil, the overall prevalence of HPV infection among women, not stratified by cervical cytology results, ranged from 16.8% to 28.6% (Ayres and Silva, 2010). In men, HPV can be present in up to 72% of the samples of the male genital region, as was reported in a study on a population of southeastern Brazil (Freire*et al.*, 2011). To our knowledge, the only study of the potential association between HPV and prostate cancer in Brazil was performed bySilvestre *et al.* (2009), in which the authors reported a 3% prevalence of HPV DNA in samples of prostatic carcinoma in a northern Brazilian population. In the northeastern region of Brazil, data on HPV prevalence are scarce, both in females and in males. On the other hand, in a study in this population, Silva Jr and da Silva (2011) investigated the prevalence of HPV DNA in 79 samples of breast ductal carcinoma and reported the absence of HPV types 6, 11, 16, and 18. Therefore, the results presented here, as well as those of Silva Jr and da Silva (2011) are important reports on the prevalence of HPV in neoplastic tissues in the northeastern population of Brazil.

Some limitations of this study should be taken into consideration. Although we used a relatively large sample size (n = 104) compared to other studies (McNicol and Dodd, 1990; Anwar *et al.*, 1992; Effert *et al.*, 1992; Carozzi *et al.*, 2004; Gazzaz and Mosli, 2009; Chen*et al.*, 2011; Michopoulou *et al.*, 2014; Singh *et al.*, 2015), the lack of association observed in this study may have been a result of the limited number of samples analyzed. Moreover, it would be useful to use complementary approaches for the direct and indirect detection of HPV to confirm our negative results. However, although serologic analysis is often used in combination with molecular analysis (Chen *et al.*, 2011, Tachezy *et al.*, 2012), this method is limited in that it reflects overall exposure to HPV and does not distinguish HPV infections within specific anatomical sites (Chen *et al.*, 2011). The molecular technique used in this study (nested PCR) is one of the most sensitive for the detection of HPV DNA in clinical specimens when combined with the sequencing of positive samples, as previously included in the study design (Chaiwongkot *et al.*, 2007). The set of primers used and the quality of DNA analyzed are the main variables that may cause errors when attempting to detect HPV DNA by PCR (Terris*et al.*, 1997). However, the set of primers used in this study is well established in the literature, and amplification of the*MDM2* gene revealed that 100% of the samples had sufficient high quality DNA for subsequent reactions.

In conclusion, our study showed no evidence to support the hypothesis that HPV infection is related to the development of prostate cancer in patients from northeastern Brazil, given that the prevalence of these pathogens in the population studied was null. Additional studies involving larger sample size and that take into account other confounding variables such as sexual behavior should be carried out to elucidate the role of HPV in prostatic carcinogenesis.

## Supplementary Material

The following online material is available for this article:

Figure S1: Agarose gel electrophoresis of total DNA extracted from prostate tissue.

Figure S2: Amplification of the DNA extracted from prostate tissue.

This material is available as part of the online article from http://www.scielo.br/gmb
