# Supplementary material for: Lack of detection of human papillomavirus DNA in prostate carcinomas in patients from northeastern Brazil
Source: Genet Mol Biol. 2016 Jan-Mar;39(1):24–9. doi: 10.1590/1678-4685-GMB-2015-0122 (PMC4807381; doi:10.1590/1678-4685-GMB-2015-0122)
Supplement: Supplementary file 1 [file 1415-4757-gmb-39-1-24-Suppl01.pdf]

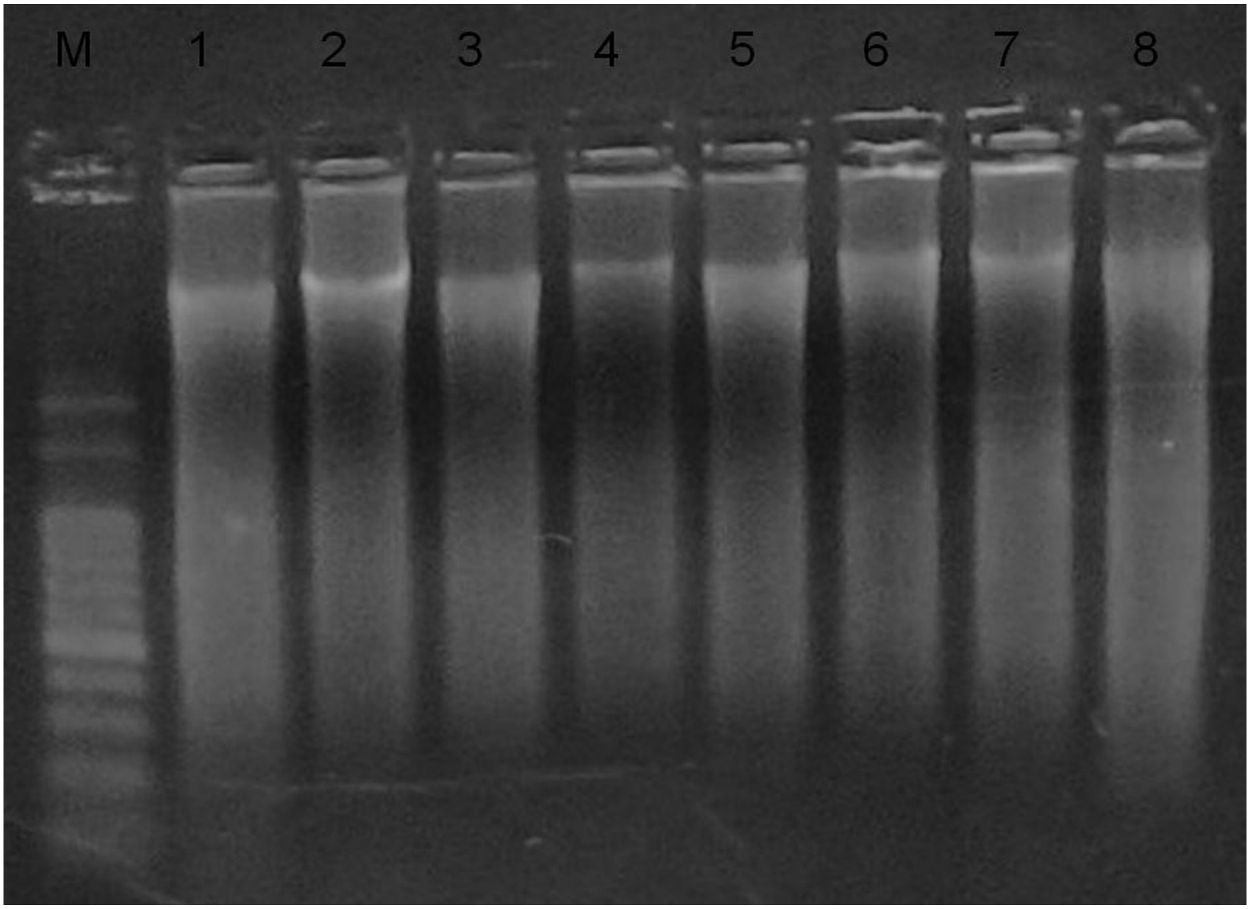

Figure S1 - Agarose gel electrophoresis (0.8%) of total DNA extracted from prostate tissue (lines 1-8). M, 100 bp ladder.
