# Supplementary material for: Lack of detection of human papillomavirus DNA in prostate carcinomas in patients from northeastern Brazil
Source: Genet Mol Biol. 2016 Jan-Mar;39(1):24–9. doi: 10.1590/1678-4685-GMB-2015-0122 (PMC4807381; doi:10.1590/1678-4685-GMB-2015-0122)
Supplement: Supplementary file 2 [file 1415-4757-gmb-39-1-24-Suppl02.pdf]

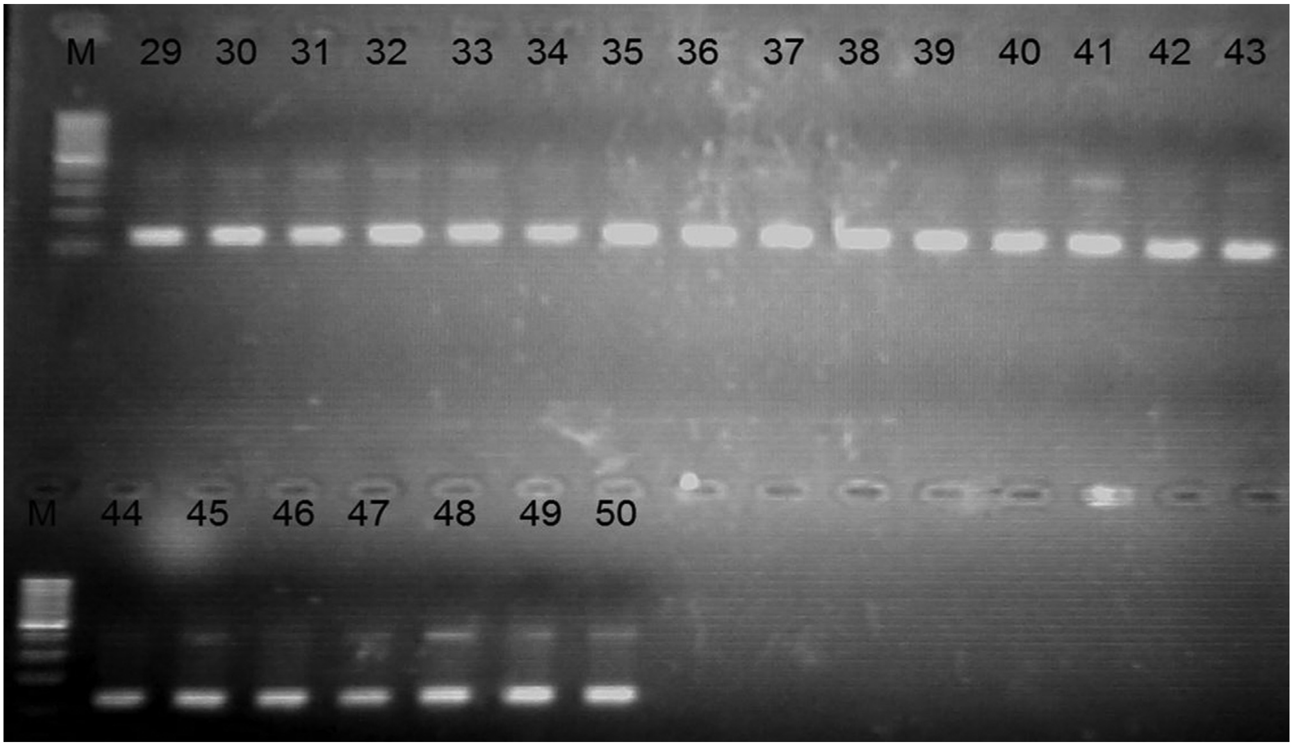

Figure S2 - Amplification results of DNA extracted from prostate tissue using *MDM2* gene primers separated by (121 bp, lines 29-50) separated by agarose gel electrophoresis (2%). M, 100 bp ladder.
